# Endothelial Dysfunction in Rheumatoid Arthritis: Mechanistic Insights and Correlation with Circulating Markers of Systemic Inflammation

**DOI:** 10.1371/journal.pone.0146744

**Published:** 2016-01-13

**Authors:** Perle Totoson, Katy Maguin-Gaté, Maude Nappey, Daniel Wendling, Céline Demougeot

**Affiliations:** 1 EA 4267 FDE, FHU INCREASE, Univ. Bourgogne Franche-Comté, Besançon, France; 2 Service de Rhumatologie, CHRU Besançon, Besançon, France; 3 EA 4266, Univ. Bourgogne Franche-Comté, Besançon, France; University of Iowa, UNITED STATES

## Abstract

**Objectives:**

To determine mechanisms involved in endothelial dysfunction (ED) during the course of arthritis and to investigate the link between cytokines, chemokines and osteoprotegerin.

**Approach and Results:**

Experiments were conducted on aortic rings at day 4 (preclinical), day 11 (onset of disease), day 33 (acute disease) and day 90 (chronic disease) after adjuvant-induced arthritis (AIA) in Lewis rats. At day 4, the unique vascular abnormality was a reduced norepinephrine-induced constriction. At day 11, endothelial function assessed by the relaxation to acetylcholine was normal despite increased cyclo-oxygenase-2 activity (COX-2) and overproduction of superoxide anions that was compensated by increased nitric oxide synthase (NOS) activity. At day 33, ED apparition coincides with the normalization of NOS activity. At day 90, ED was only observed in rats with a persisting imbalance between endothelial NOS and COX-2 pathways and higher plasma levels of IL-1β and TNFα. Plasma levels of IL-1β, TNFα and MIP-1α negatively correlated with Ach-induced relaxation throughout the course of AIA.

**Conclusions:**

Our data identified increased endothelial NOS activity as an important compensatory response that opposes the ED in the early arthritis. Thereafter, a cross-talk between endothelial COX-2/NOS pathways appears as an important element for the occurrence of ED. Our results encourage determining the clinical value of IL-1β, TNFα and MIP-1α as biomarkers of ED in RA.

## Introduction

Rheumatoid arthritis (RA) is the most common systemic autoimmune disease resulting in excessive cardiovascular (CV) events and mortality [1]. The CV risk is roughly 2-fold that of the general population and was recently found to be comparable to the risk in diabetes [2]. Increased incidence of CV mortality is mainly the consequence of accelerated atherogenesis which is secondary to endothelial dysfunction (ED) [3], a vascular abnormality present in RA patients [4]. Therefore the understanding of mechanisms involved in ED as well as their time-course in RA is an important challenge for designing the adequate therapeutic strategy to reduce CV risk. A few animal studies provided mechanistic data on ED using the widely-used model of adjuvant-induced arthritis (AIA) in rats. Our systematic literature review reported increased cyclo-oxygenase-2 (COX-2) activity, overproduction of superoxide anions (O_2_^.-^) and nitric oxide synthase (NOS) uncoupling as seminal mechanisms involved in ED, at least in the acute inflammatory phase of AIA [5]. Recently, we reported that ED was absent in the early symptomatic stages of the disease but became detectable at the time of maximal inflammation in AIA [6]. However, ED is not a dichotomous parameter (“present” or “not present”) and develops over a long period of time. Thus the time at which changes in endothelial phenotype appear during the course of arthritis is not known whereas its knowledge may help to identify the “window of opportunity” for CV risk prevention in RA. In addition, whether these phenotypes changes persist when disease activity decreased is unknown.

ED is considered as the consequence of a combination of elements including genetic predisposition, traditional CV risk factors and systemic inflammation [7]. This latter factor is suspected to be a pivotal contributor of ED in RA because pro-inflammatory mediators such as IL-1β and TNF-α, that might putatively modify endothelial phenotype, are produced by the inflamed synovium and released into circulation [8]. The above pro-inflammatory factors up-regulate the expression of adhesion molecules by endothelial cells and stimulated the release of chemokines. Therefore it is striking that in RA the contribution of systemic mediators of inflammation to ED is not yet elucidated [9–11]. Likewise whether such circulating indices of systemic inflammation could be used as biomarkers of ED is not demonstrated.

The first aim of this study was to investigate the mechanisms of ED from the preclinical to the late stages of the disease in the model of adjuvant-induced arthritis (AIA) in rats with an emphasis on the role of NOS, COX-2 and O_2_^.-^. The second aim was to explore the potential value of various circulating cytokines/chemokines to reflect ED.

## Material and Methods

### Animals

Six-week-old male Lewis rats (n = 160) were purchased from Janvier (Le Genest Saint Isle, France). Animals were kept under a 12h-12h light: dark cycle and allowed free access to food and water. This work is the extended study for our previous report [6] and all experimental procedures were approved by the local committee for ethics in animal experimentation n° 2012/001-CD) of Franche-Comté University (Besançon, France), and complied with the Guide for the Care and Use of Laboratory Animal published by the US National Institutes of Health (NIH publication No. 85–23, revised 2011).

### Induction and clinical evaluation of the arthritis model

As previously described [6], adjuvant arthritis was induced by a single intradermal injection at the tail of Lewis rats, of 120 μL of 1 mg of heat-killed *Mycobacterium butyricum* (Difco, Detroit, MI) suspended in 0.1 ml of mineral oil (Freund’s incomplete adjuvant (Difco, Detroit, MI)). A group of saline-infused non-arthritic rats was used as controls. Rats were weighed and monitored 5 days per week for clinical signs of arthritis. The scoring system was employed as follows [12]: arthritis of one finger scores 0.1, weak and moderate arthritis of one big joint (ankle or wrist) scores 0.5 and, intense arthritis of one big joint scores 1. Tarsus and ankle were considered as the same joint. Sum of joints scores of 4 limbs leads to an arthritic score of maximum 6 to each rat. Rats were anaesthetized with pentobarbital (60 mg/kg, i.p.) at different days after inoculation with *Mycobacterium* or saline corresponding to different stages of the disease [13]: *day 4* that represents the preclinical expression of arthritis and a time during antigen processing; *day 11* that represents the clinical onset of arthritis (very early arthritis); *day 33* corresponding to severe active disease with severe joint swelling and destruction associated to macrovascular ED; *day 90* that represents a time point at which the disease has been resolved, based on a lack of inflammation in the affected joints. Blood was withdrawn from the abdominal artery, immediately centrifuged at 3000*g* for 10 min at 4°C, and plasma was stored at -80°C until analysis. Thoracic aorta were removed and immediately used for vascular reactivity studies. Rats were sacrificed by exsanguination during this procedure.

### Radiographical ex-vivo analysis of joints of ankle and foot

Radiographs of hind paws were performed at each stage of arthritis with a BMA High Resolution Digital X Ray (40mV, 10mA)–D3A Medical Systems (France). A score of 0 to 20 was determined for each paw using a grading scale modified from Ackerman et al. (1979) [14]. This score used the scale: 0 (normal), 1 (slight), 2 (mild), 3 (moderate), and 4 (severe) abnormalities in the tissue for each of 5 characteristic features of AIA. Radiographs takes into account: (a) the soft tissue swelling, (b) the osteoporosis as measured by bone density, (c) the loss of cartilage shown by narrowing of the joint spaces; (d) the bone erosions and (e) the heterotopic ossification defined as proliferation of new bone tissue. The maximum score for each rat is 40.

### Vascular reactivity

At each time of arthritis course, thoracic aorta was excised, cleaned of connective tissue, and cut into rings of approximately 2 mm in length. Rings were suspended in Krebs solution (NaCl 118 mmoles/liter, KCl 4.65 mmoles/liter, CaCl_2_ 2.5 mmoles/liter, KH_2_PO_4_ 1.18 mmoles/liter, NaHCO_3_ 24.9 mmoles/liter, MgSO_4_ 1.18 mmoles/liter, glucose 12 mmoles/liter, pH 7.4), maintained at 37°C and continuously aerated with 95% O_2_, 5% CO_2_, for isometric tension recording in organs chambers, as previously described [15]. In some rings, endothelium was mechanically removed. The completeness of endothelial denudation was confirmed by the absence of relaxation to the endothelium-dependent agonist acetylcholine (ACh, 10^−6^ moles/liter). After a 90-min-equilibration period under a resting tension of 2g, rings were exposed to high extracellular KCl (100 mmoles/liter) to measure a strong tonic contractile response (expressed in g). After washing, endothelium-denuded rings were used to determine the vasoconstrictive response to norepinephrine (NE, 10^−11^–10^−4^ moles/liter) and to evaluate the endothelium-independent relaxation to the NO donor sodium nitroprussiate (SNP, 10^−11^–10^−4^ moles/liter) after preconstriction with PE 10^−6^ moles/liter. In parallel rings with intact endothelium were constricted with phenylephrine (PE, 10^−6^ moles/liter) and endothelium-dependent relaxation was assessed with Ach (10^−11^–10^−4^ moles/liter). The results regarding the effect of Ach-induced relaxation in AIA and controls have been presented elsewhere [6]. To dissect the mechanisms involved in endothelial response to Ach, rings were incubated for 30-min either with the non-selective NO synthase inhibitor N_W_-nitro-L-arginine methyl ester (L-NAME, 10^−4^ moles/liter), the selective COX-2 inhibitor (NS398, 10^−5^ moles/liter), and the superoxide dismutase mimetic (SOD) Tempol (10^−4^ moles/liter). Contractile responses to NE were calculated as the percentage of the maximum response to high KCl, and vasorelaxant responses to ACh and SNP were calculated as the percentage of relaxation of PE-induced contraction.

### Plasma measurements

Plasma levels of pro-inflammatory cytokines (interleukin 1β (IL-1β) and Tumor Necrosis Factor α (TNFα)), anti-inflammatory cytokines (interleukin 4 (IL-4) and interleukin 10 (IL-10)), monocyte/macrophage chemokines (Macrophage Inflammatory Protein (MIP-1α), Monocyte Chemoattractant Protein (MCP-1)) and osteoprotegerin (OPG) were measured by using Milliplex magnetic bead panel kits (eBioscience, Vienne, Austria) that were analyzed using a Luminex MAGPIX system (Luminex Corporation; Houston, TX) and Milliplex Analyst software (Millipore; St. Charles, MO). The limits of detection provided by the manufacturer for IL-1β, TNFα, IL-4, IL-10, MIP-1α, MCP-1 and OPG were (pg/mL) 13, 3.78, 0.82, 8.59, 2.32, 18 and 1 respectively.

#### Data and statistical analysis

Values are presented as means ± SEM. Data were analyzed using GraphPad Prism software (version 5.0). The overall relaxant response to ACh and the constrictive response to NE were characterized by the area under the curve (AUCs) calculated from the individual concentration-response curves. Concentration-responses to ACh in the presence or not of a specific inhibitor were compared by 2-way analysis of variance (ANOVA) for repeated measures. To better understand the effect of L-NAME in AIA as compared to control rats, the results were expressed as the percentage of reduction of AUCs between curves of Ach without and with L-NAME. Comparison of two values between AIA and controls was assessed by using unpaired Student *t* test or Mann-Whitney test when data were not normally distributed. Analysis of the relationship between two parameters was determined by linear regression analysis and Spearman’s correlation coefficient was calculated between these variables. P<0.05 was considered statistically significant.

## Results

### Time-course of clinical and radiographic parameters in AIA rats

Consistent with our earlier study [6], the first clinical signs of arthritis (paw edema, erythema, stiffness) appeared between day 11 and day 14 after immunization. From day 11 onwards, the severity of arthritis increased until day 33. After the period of acute clinical inflammation, clinical signs of inflammation spontaneously regressed but joint malformations are still evident. Consistent with the progression of bone damage in AIA, radiographic score progressively increased from day 4 to day 90. The means arthritis scores and radiographic score at different phases of AIA are presented in Table 1.

**Table 1:** Clinical, radiological characteristics and cytokines/chemokines levels.

		Day post-immunization			
		4	11	33	90
Arthritis score	AIA	0	1.9 ± 0.1	2.5 ± 0.2	1.1 ± 0.2
Radiographic score	AIA	0.8 ± 0.5	3.1 ± 0.6	21.6 ± 2.1	21.9 ± 0.7
IL-1β	Controls	23.3 ± 4.3	15.8 ± 2.9	9.6 ± 3.2	8.7 ± 1.1
	AIA	38.3 ± 5.5	49.7 ± 6.1*	83.4 ± 13.5*	46.8 ± 9.1*
TNF-α	Controls	6.7 ± 1.1	3.8 ± 0.7	2.7 ± 0.7	2.4 ± 0.3
	AIA	7.0 ± 1.1	5.4 ± 0.7	18.0 ± 3.1*	11.9 ± 2.3*
IL-10	Controls	12.7 ± 1.3	12.7 ± 0.9	13.8 ± 2.5	13.4 ± 1.5
	AIA	21.8 ± 2.5*	30.6 ± 3.4*	16.6 ± 1.6	12.5 ± 0.9
IL-4	Controls	0.3 ± 0.1	0.3 ± 0.1	0.3 ± 0.1	0.4 ± 0.1
	AIA	0.5 ± 0.1	0.4 ± 0.0	0.4 ± 0.0	0.4 ± 0.1
MIP-1α	Controls	13.2 ± 1.7	9.1 ± 1.1	14.6 ± 4.0	6.1 ± 0.8
	AIA	14.6 ± 2.2	11.6 ± 1.5	31.5 ± 4.7*	17.4 ± 2.9*
MCP-1	Controls	1580 ± 155	1365 ± 114	1138 ± 105	915 ± 64
	AIA	2612 ± 208*	3693 ± 315*	2046 ± 162*	1182 ± 113
OPG	Controls	224 ± 19	230 ± 15	169 ± 15	162 ± 12
	AIA	262 ± 18	537 ± 48*	212 ± 16	187 ± 35

Clinical data were obtained from all rats (n = 20/group/stage). Plasma cytokines and chemokines levels (pg/mL) measured at day 4, 11, 33 and 90 post-immunization in AIA and age-matched controls (n = 11–17 per AIA groups and n = 7–12 per Control groups). Values are the mean ± SEM.

*p <0.05 versus age-matched control.

### Profile of plasma cytokines, chemokines and OPG levels during AIA

Data are presented in Table 1. Except IL-4 levels that were not changed by AIA, all cytokines/chemokines increased during AIA but with a different profile. As compared to controls, IL-10 levels increased in AIA at day 4 and day 11 but not thereafter. MCP-1 levels were increased in AIA from day 4 to day 33 but not at day 90. IL-1β levels were higher in AIA from day 11 to day 90. The increase in TNFα and MIP-1α levels occurred later, at day 33 and day 90. OPG was significantly elevated at day 11 but not at the other times.

### Vascular contractile function in AIA

**Fig 1:**
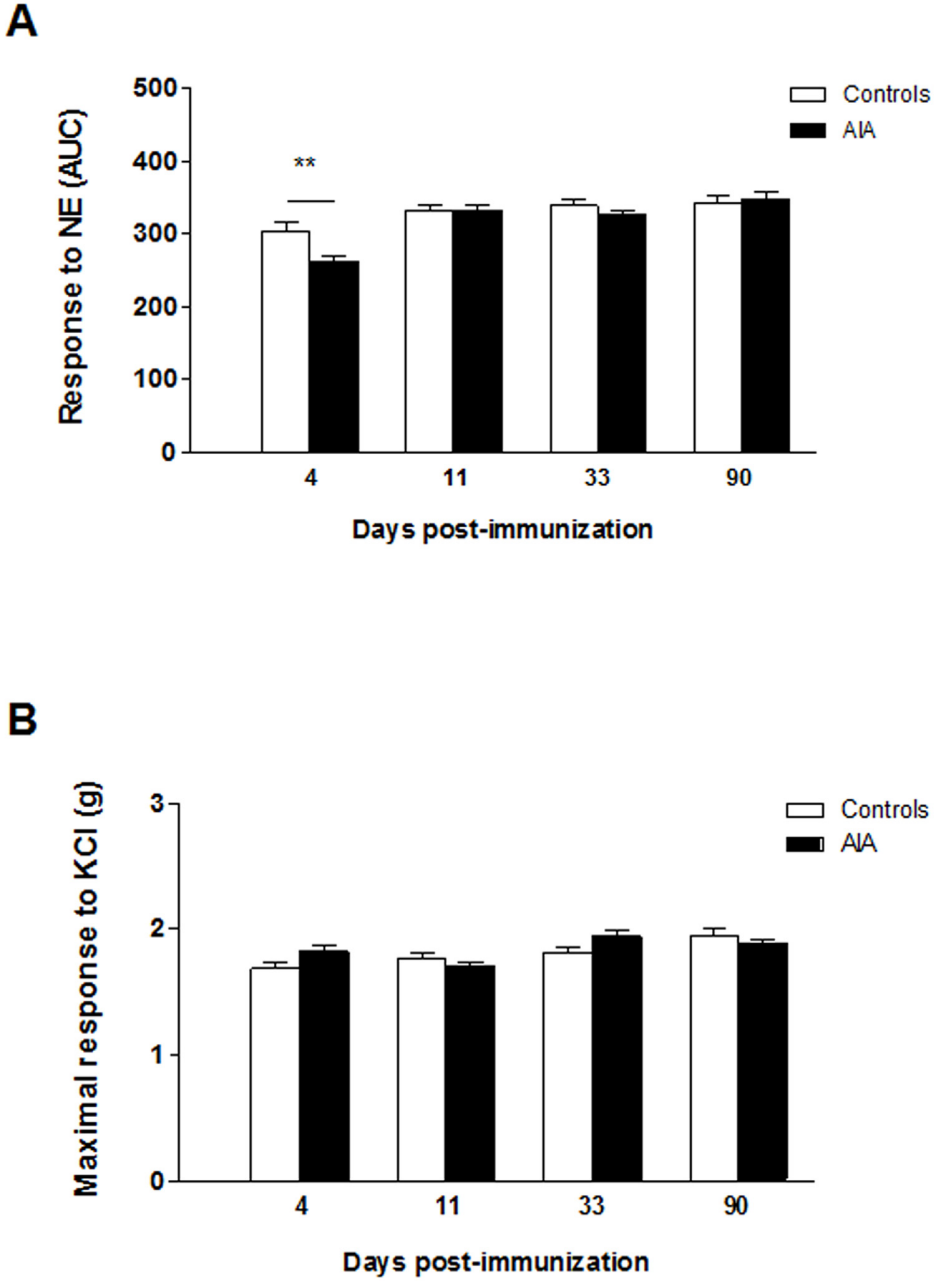
Vascular contractile function during the course of AIA. The vasoconstrictive response to norepinephrine (NE, 10^−11^–10^−4^ moles/liter) (A) and to 100 mmoles/liter of KCl (B) was assessed on endothelium-denuded aortic rings. Experiments were performed from AIA and control rats at each stage of arthritis. The overall response to norepinerphine is expressed as area under the curve (AUC) whereas the response to KCl was expressed as the maximal contraction (expressed in g). Values are means ± SEM of 12 to 20 aortic rings from 12 to 20 rats/group/stage. **p< 0.01 vs controls.

Before investigating endothelial function and related pathways, we checked whether differences exist regarding the response of vascular smooth muscle cells (VSMCs) to NE or KCl. As shown in Fig 1A, as compared to controls, AIA exhibited reduced constrictive response to NE at day 4 post-immunization (p<0.01) but not thereafter. By contrast, no significant difference in maximal response to KCl was observed between the two groups whatever the stage of arthritis.

### Endothelial NOS, COX-2 and superoxide anion pathways during the course of AIA

To determine at which time of the arthritis course abnormalities in endothelial pathways appear, the effects of the NOS inhibitor L-NAME, the COX-2 inhibitor NS-398 and the SOD mimetic Tempol were studied in Ach-relaxed rings from AIA and controls rats.

**Fig 2:**
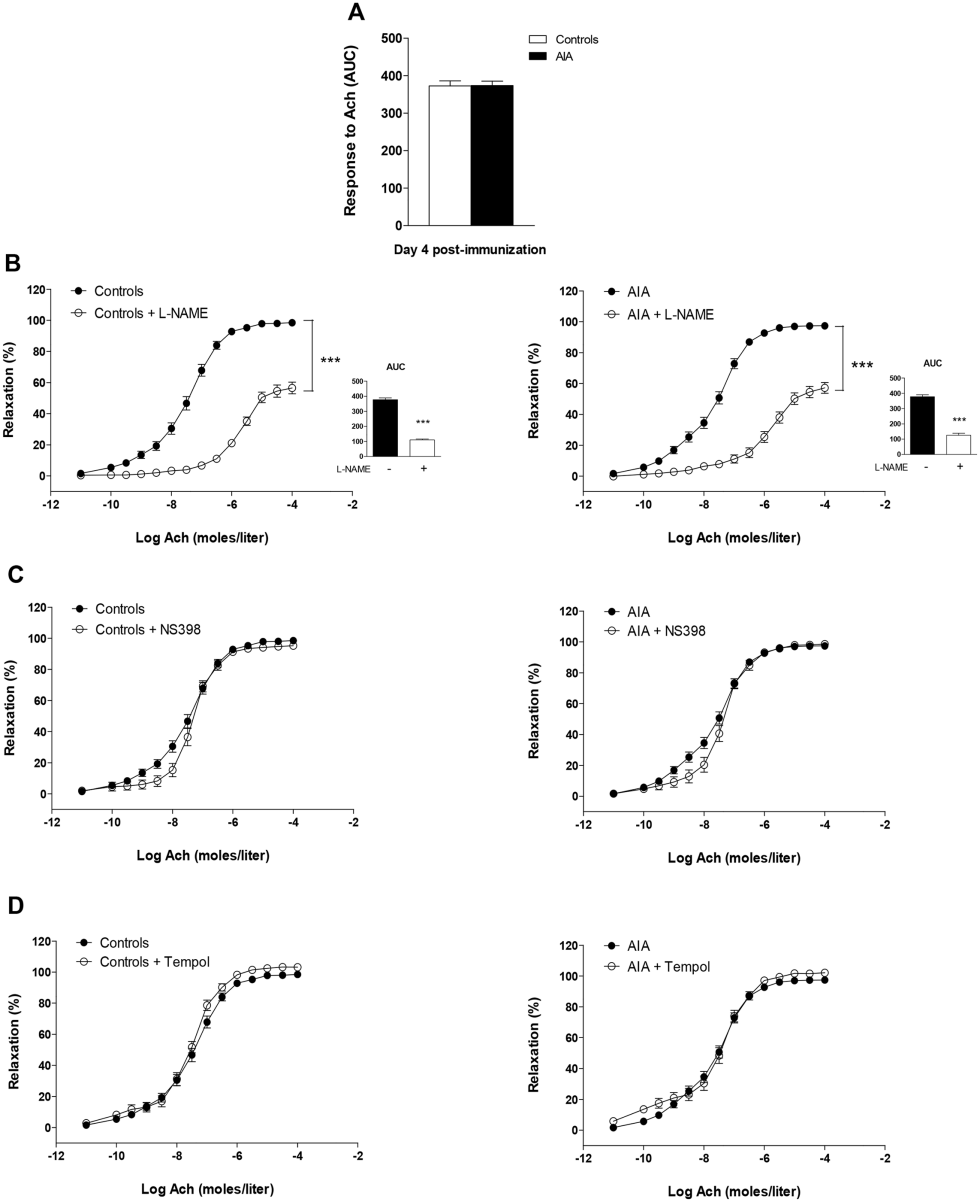
Effect of L-NAME, NS398 and Tempol on the response to Ach at the preclinical stage of AIA. (A) The vasodilator response to Ach (expressed as AUC of Ach) was assessed in aortic rings from AIA and controls at day 4 post-immunization. The same experiment was performed after incubation with L-NAME (B) (the insert presents AUC of Ach), NS398 (C) and Tempol (D). Results are expressed as means ± SEM of 13 to 17 aortic rings from 17 rats/group. *** p<0.001.

At day 4, Ach-induced relaxation was not impaired [6] (Fig 2A). Neither NS-398 nor Tempol modified Ach-induced relaxation in controls or in AIA thus indicating a lack of deleterious COX-2 activity and O_2_^.-^ production at this time (Fig 2C–2D). L-NAME significantly blunted Ach-associated relaxation in both groups, to the same extent (% reduction of AUC: 69 ± 10 vs 70 ± 2 in controls, NS) (Fig 2B). This indicates no difference in NOS activity between groups.

**Fig 3:**
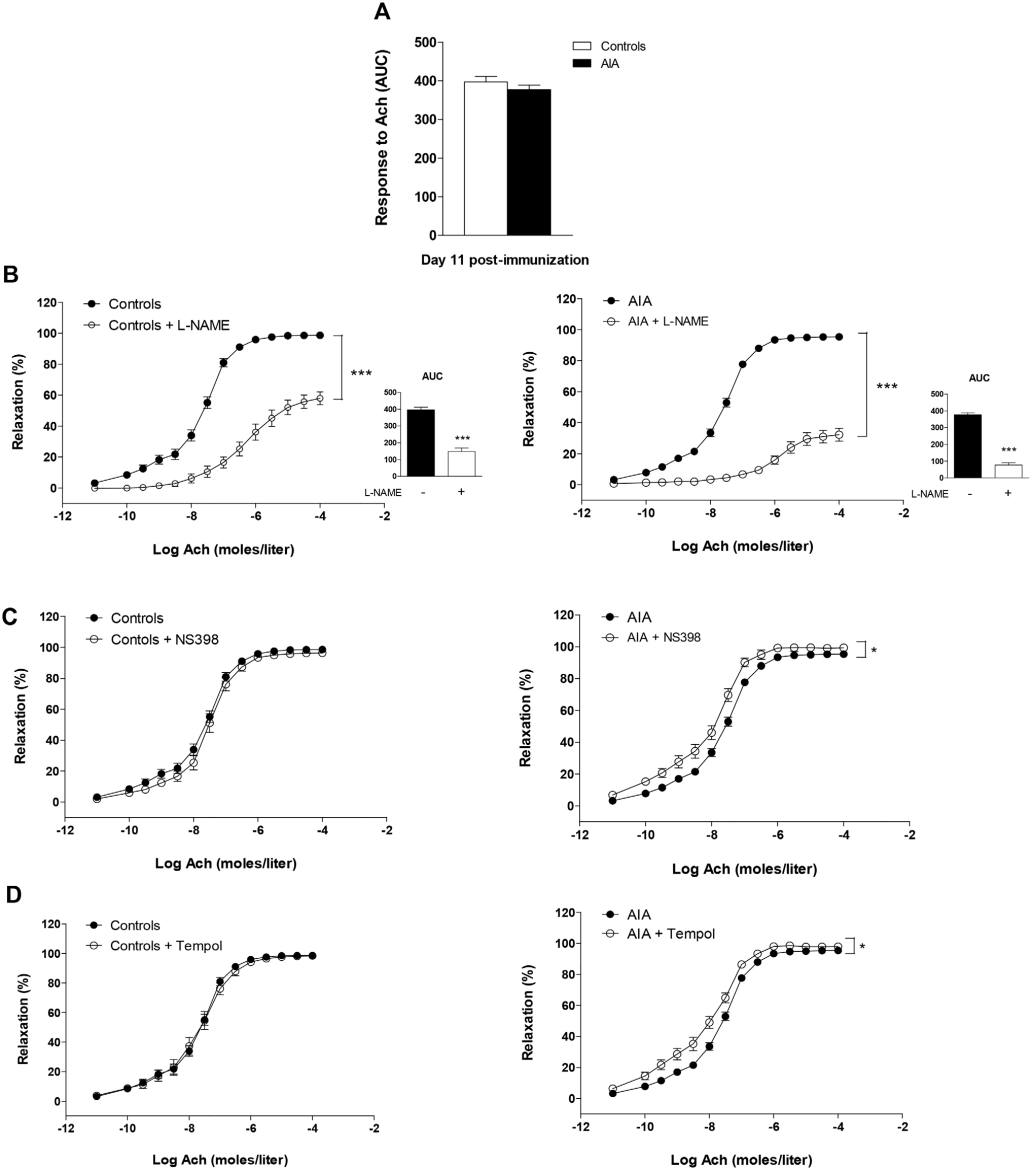
Effect of L-NAME, NS398 and Tempol on response to Ach at the clinical onset of arthritis. (A) The vasodilator response to Ach (expressed as AUC of Ach) was assessed in aortic rings from AIA and controls at day 11 post-immunization. The same experiment was performed after incubation with L-NAME (B) (the insert presents AUC of Ach), NS398 (C) and Tempol (D). Results are expressed as means ± SEM of 12 to 18 aortic rings from 18 rats/group. *p<0.05, *** p<0.001.

At day 11 (Fig 3A), endothelial function was normal in AIA rats [6]. However, as a reflection of enhanced NOS activity in AIA, the reduction of AUC of Ach induced by L-NAME was significantly greater in AIA (80 ± 4%) than in controls (61 ± 8%, p<0.05, Fig 2B). Of interest, NS-398 (Fig 3C) and Tempol (Fig 3D) slightly but significantly improved Ach-induced vasodilation in AIA but not in controls. These data revealed that abnormal overactivation of COX-2 associated with O_2_^.-^ production was initially compensated by increased NOS activity.

**Fig 4:**
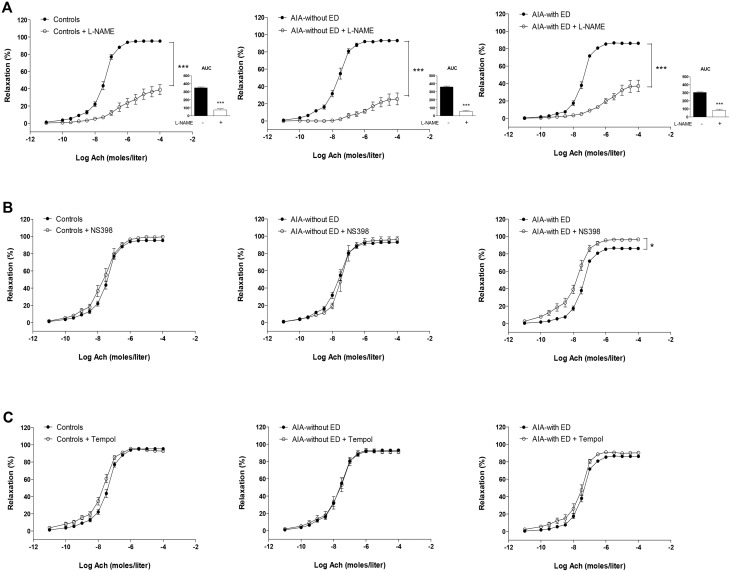
Effect of L-NAME, NS398 and Tempol on response to Ach at the acute inflammatory phase of AIA. (A) The vasodilator response to Ach (expressed as AUC of Ach) was assessed in aortic rings from AIA and controls at day 33 post-immunization. The same experiment was performed after incubation with L-NAME (B) (the insert presents AUC of Ach), NS398 (C) and Tempol (D). Results are expressed as means ± SEM of 12 to 19 aortic rings from 19 rats/group. ** p<0.01, *** p<0.001.

At day 33, an impaired response to Ach was observed in AIA [6] (Fig 4A). The dysfunctions regarding COX-2 and O_2_^.-^ in AIA observed at day 11 were still evident (Fig 4C–4D) but, as shown in Fig 4B, NOS activity is no longer increased in AIA as attested by the lack of difference in the effect of L-NAME between AIA and controls (% reduction of AUC: 80 ± 4 in AIA vs 72 ± 5 in controls, NS).

**Fig 5:**
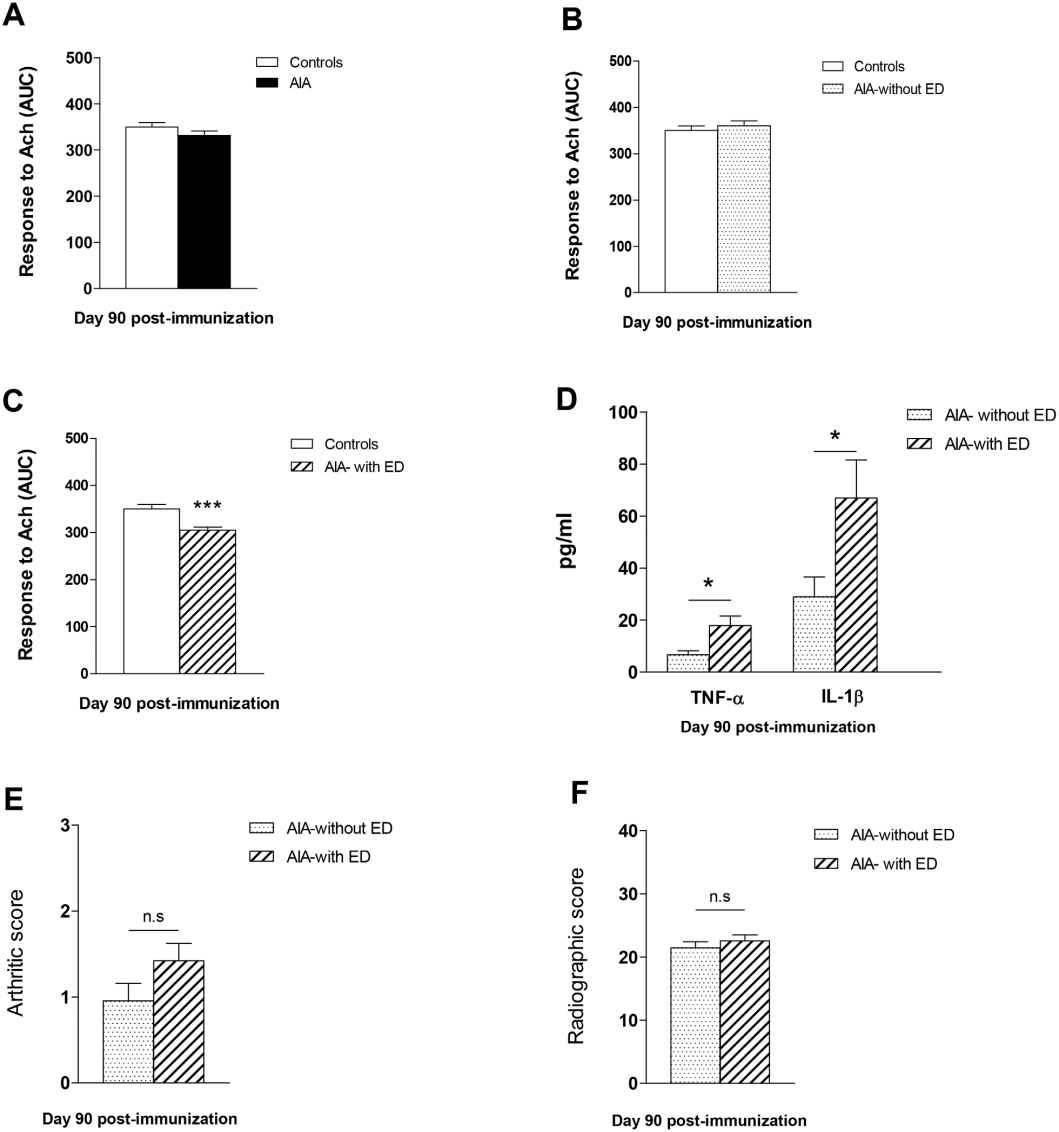
Endothelial function and cytokines levels at day 90 post-immunization. Experiments were performed from AIA and control rats at day 90 post-immunization. (A) The overall vasodilator response to Ach (expressed as AUC of Ach) was not different in AIA vs control rats. (B,C) The detailed analysis of individual data revealed two populations of AIA rats, with and without endothelial dysfunction. These two groups differed from pro-inflammatory cytokines levels (D) but not from arthritis score (E) or radiographic score (F). Values are as means ± SEM from 8 to 11 values/group. *p< 0.05, p*** p<0.001.

**Fig 6:**
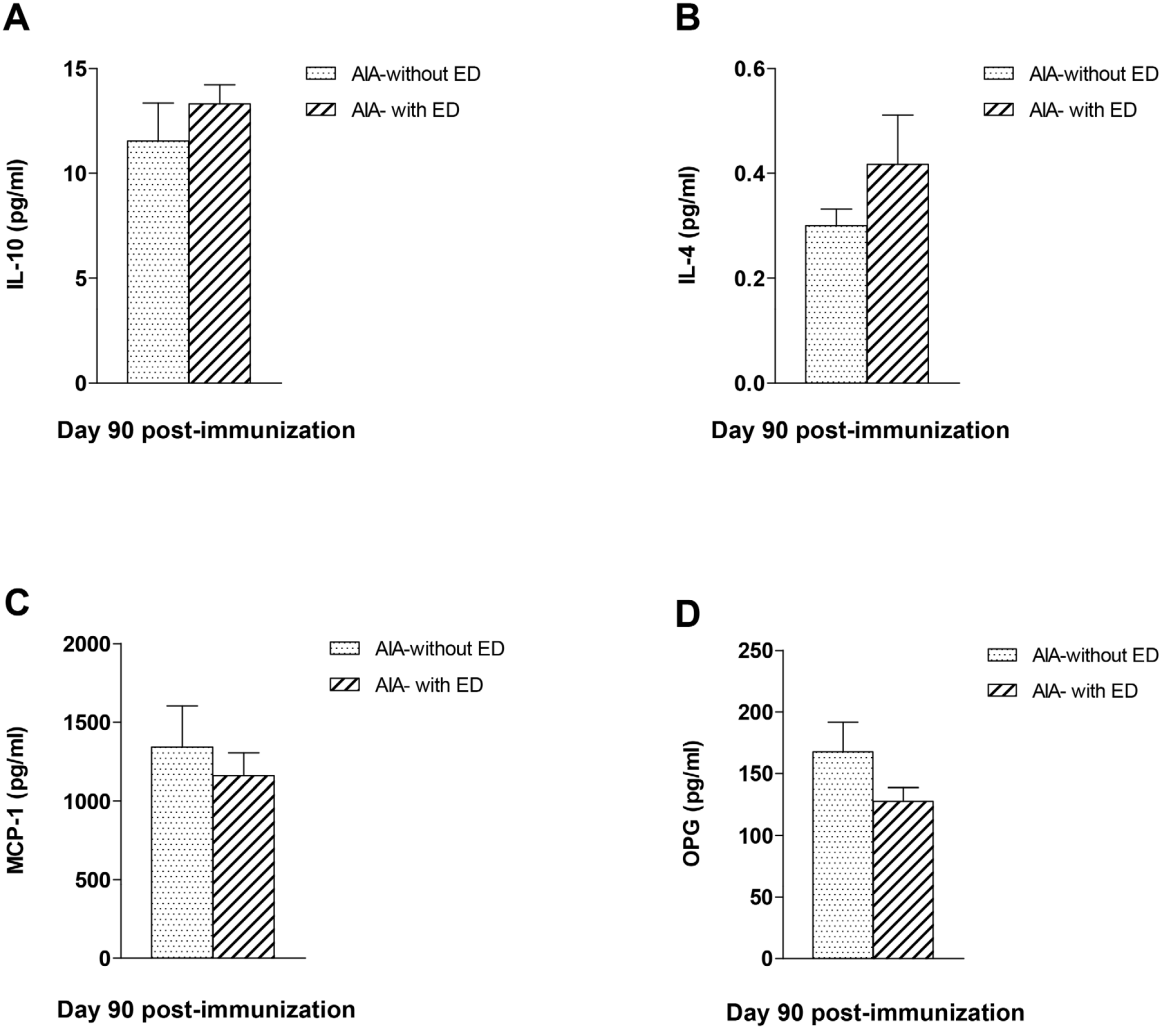
Plasma levels of IL-10, IL-4, MCP-1 and OPG in AIA rats at day 90 post-immunization. At day 90 post-immunization, plasma levels of IL-10 (A), IL-4 (B), MCP-1 (C) and OPG (D) did not differ between AIA rats “with endothelial dysfunction (ED)” and AIA “without ED”. Results are expressed as means ± SEM from 6 to 9 rats/subgroups.

**Fig 7:**
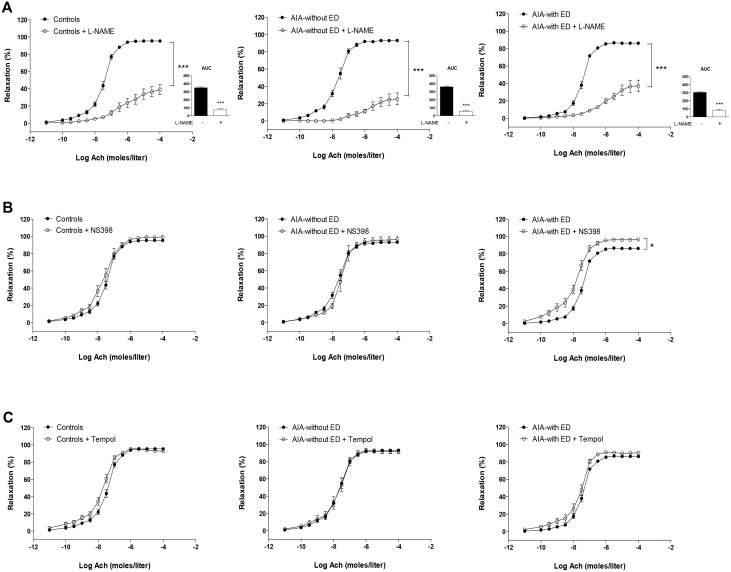
Effect of L-NAME, NS398 and Tempol on vasodilation response to Ach at the late phase of arthritis. Cumulative concentration curves were performed in aortic rings from controls, AIA without endothelial dysfunction (ED) and in AIA with ED, in the presence or not of L-NAME (A) (the insert presents AUC of Ach), NS398 (B) and Tempol (C). Values are as means ± SEM of 6 to 11 aortic rings from 15 Controls rats, 11 rats AIA-without ED and 9 rats AIA-with ED. *p< 0.05, *** p<0.001.

At day 90, the overall response of AIA to Ach was not different from controls [6] (Fig 5A). However, the detailed analysis of individual data revealed that two populations of AIA emerged on the basis of their response to Ach. One group (55% of rats, “without ED”) did not exhibit impaired response to Ach (Fig 5B), whereas another group (45% of rats, “with ED”) had depressed response to Ach (p<0.001, Fig 5C). Of interest, differences in cytokines levels were found between these 2 groups of rats at day 90. As compared to the AIA group without ED, the AIA group with ED had higher levels of TNFα and IL-1β (Fig 5D, p<0.05) whereas no difference was found for the other cytokines or chemokines (Fig 6). By contrast, arthritis (Fig 5E) and radiographic scores (Fig 5F) did not differ between these two populations. As shown in Fig 7A, in AIA “with ED”, the effect of L-NAME was not different from controls (% reduction of AUC: 72 ± 4 vs 75 ± 5 in controls, NS) whereas its effect was greater in AIA “without ED” compared to controls, thus reflecting enhanced NOS activity in this latter group (% reduction of AUC: 84 ± 4 vs 75 ± 5 in controls, p<0.05). Likewise, a difference in COX-2 activity was observed between AIA “with ED” and “without ED”. In the former, NS-398 still improved the vasorelaxant effect of Ach as a reflection of persisting deleterious COX-2 activity whereas this inhibitor was devoid of impact on Ach-relaxation in the latter (Fig 7B). By contrast, Tempol did not modify the effect of Ach whatever the group (Fig 7C). Above results suggest that the presence of ED in AIA rats at the late phase of arthritis did not rely on O_2_^.-^ overproduction but on the persisting imbalance between NOS and COX-2 endothelial pathways.

### Endothelium-independent relaxation to SNP in AIA

**Fig 8:**
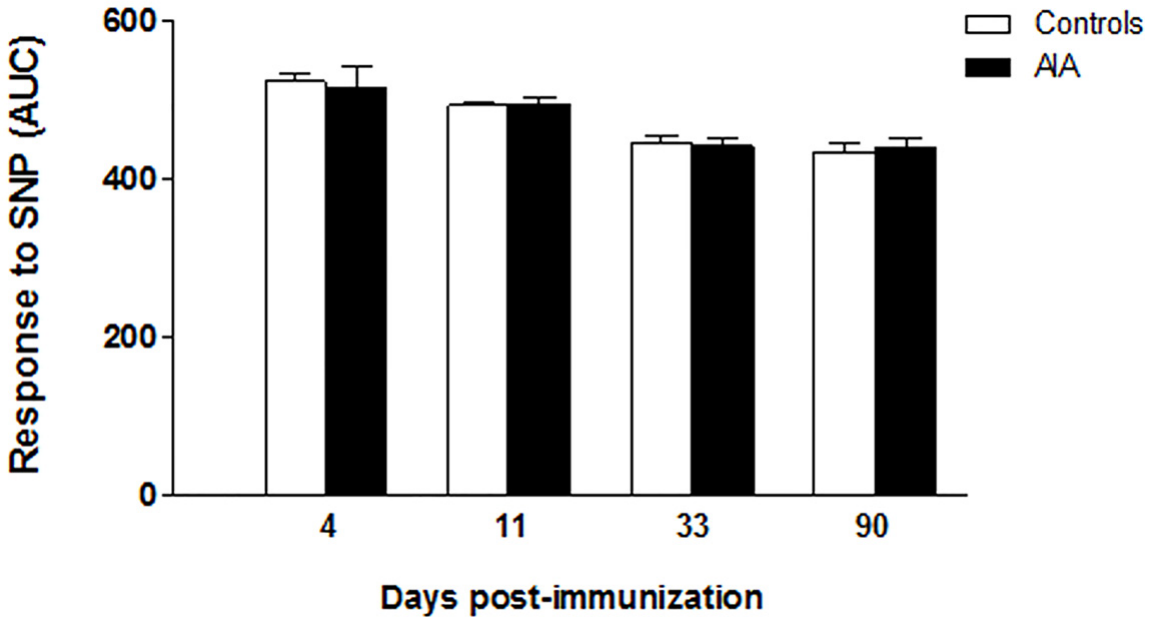
Endothelium-independent relaxation during the course of arthritis. The vasodilator response to the NO donor SNP (expressed as AUC) was assessed on endothelium-denuded aortic rings after preconstriction with PE (10^-6^moles/liter). Results are expressed as means ± SEM of 10 to 19 aortic rings from 20 rats/group/stage.

To ascertain that changes observed in the response to Ach were not due to an impaired response of VSMCs to NO, the relaxation to the NO donor SNP were compared between controls and AIA. As shown in Fig 8, the relaxing responses to SNP were not different between both groups at any stage of arthritis.

### Correlations between endothelial function and cytokines/chemokines/OPG plasma levels

**Fig 9:**
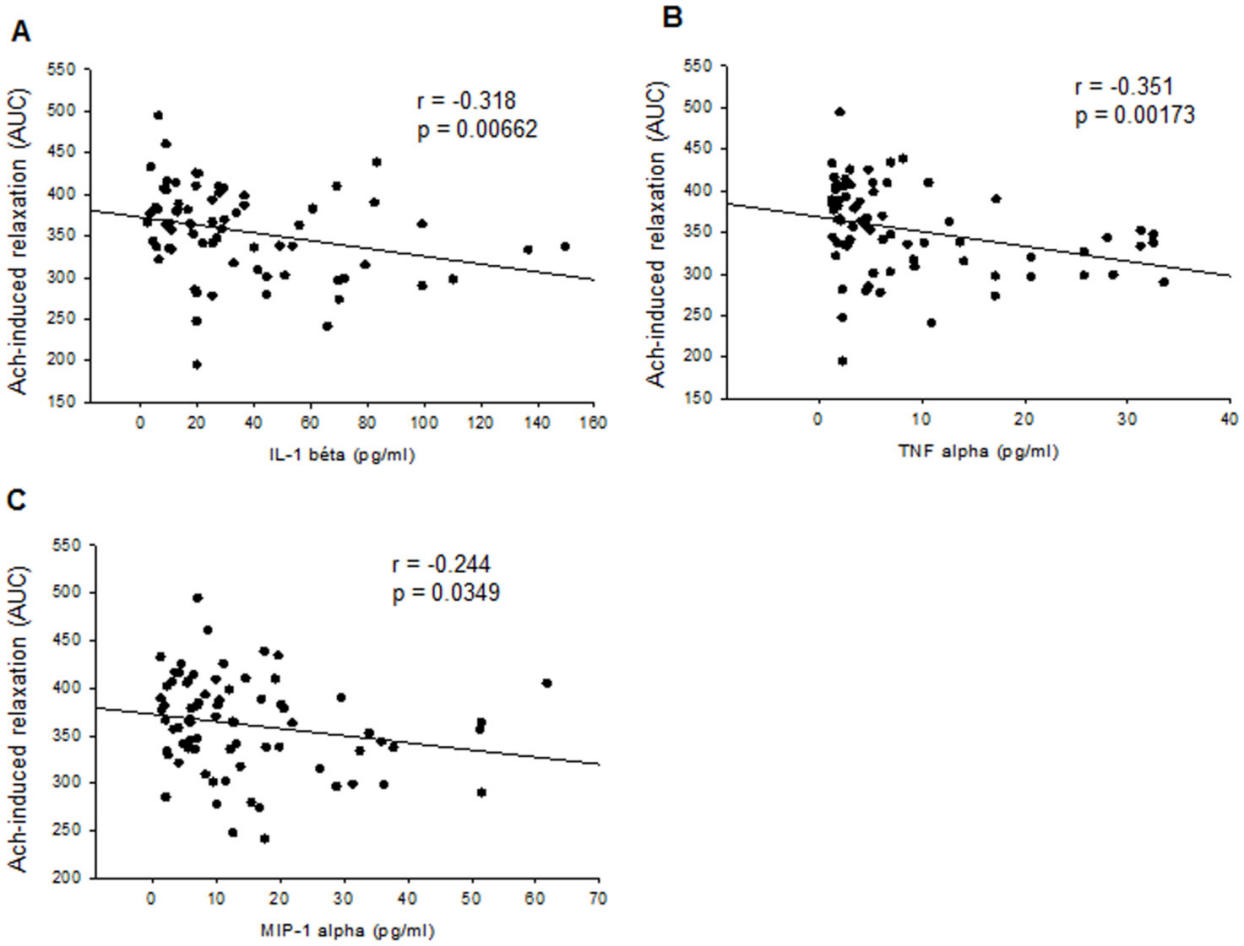
Correlations between endothelial function and cytokines/chemokines plasma levels. Ach-induced relaxation (expressed as AUC) and plasma levels of IL-1β (A), TNF-α (B) and MCP-1 levels (**C**) negatively correlated throughout the AIA course (from day 4 to day 90 post-immunization).

To determine whether levels of mediators of inflammation might be used as circulating markers of ED, we investigated the correlations between Ach-induced relaxations (expressed as AUCs) from day 4 to day 90 and the corresponding levels of cytokines/chemokines (Table 1). The results showed that plasma levels of IL-1β, TNFα and MIP-1α negatively correlated with Ach-induced relaxation (Fig 9). By contrast, no correlation was found between endothelial function and levels of IL-4 (r = -0.0561, p = 0.953), IL-10 (r = 0.0937, p = 0.441), MCP-1 (r = 0.199, p = 0.0966) and OPG (r = 0.169, p = 0.124).

## Discussion

The seminal findings of our study in AIA are that 1) increased NOS activity is an important compensatory response that opposes the ED in the early phase of the disease, 2) plasma levels of IL-1β, TNFα and MIP-1α correlate with endothelial function throughout the course of AIA, 3) impaired response of smooth muscle to vasoconstriction precedes the alterations of endothelial phenotype.

The understanding of the pathogenesis of ED in RA is a prerequisite to propose an adequate therapeutic strategy for CV risk prevention. Previous analysis of the literature identified increased COX-2 activity, O_2_^.-^ overproduction and NOS decoupling as seminal mechanisms involved in ED at the acute phase of arthritis [5]. The new findings are that these pathways are also altered in the very early phase of AIA as well as in the late phase after resolution of inflammatory symptoms. Our study demonstrated that despite the lack of detectable ED at the onset of clinical symptoms, the deleterious O_2_^.-^ production and COX-2 activity are initially compensated by increased vascular NOS activity thereby opposing ED. These findings can be related to those of a clinical study in which the basal forearm blood flow was measured in newly diagnosed RA patients in the presence or not of a NOS inhibitor [16]. The authors found a greater inhibition of blood flow by the NOS inhibitor in RA patients than in controls, which is consistent with a compensatory NOS activation in this early stage of RA. It is striking that this considerable endothelial plasticity is not specific to RA since the same compensatory NOS upregulation exists in the early phase of models of hypertension [17] or diabetes [18]. These suggest that common mechanisms are involved in such compensation. As O_2_^.-^ has been involved in NOS pathway upregulation [17] and is overproduced in hypertension or diabetes [19], its excessive production at the onset of AIA might contribute to the high NOS activity. However, our results at day 90 showed that increased NOS activity occurs without impaired O_2_^.-^ production, so this mechanism is likely of minor importance. Whatever the mechanisms involved in NOS upregulation, overall data identify NOS is a pivotal target for the early CV risk prevention in RA, and encourage the use of therapy aiming to increase NOS activity such as arginase inhibition or L-arginine supplementation.

The occurrence of ED at the acute inflammatory phase of arthritis (day 33) coincides with loss of NOS compensation and enhancement of COX-2/O_2_^.-^ impairments. Given the recognized cross-talk between COX-2 and NOS pathways [20, 21], decreased NOS activity might result from COX-2 overactivity but in turn itself enhance COX-2 activity in a vicious circle. Of interest at this time of arthritis, plasma IL-1β and MCP-1 levels are elevated in AIA like at day 11, but additionally TNF-α and MIP-1α increased. It could be hypothesized that TNF-α might contribute to the loss of NOS compensation. Indeed, whilst both TNF-α and IL-1β have been reported to acutely induce ED [22, 23], only TNF-α was reported to decrease endothelial NOS expression [24]. The putative role of IL-1β and TNF-α, the two dominant mediators of immune-mediated joint disease in RA patients and experimental animals [25], in arthritis-associated ED is further suggested by the results obtained at day 90. At this time, the rats exhibiting ED are those with the highest IL-1β and TNF-α levels. However, the present study was not designed for determining the causal link between plasma cytokines/chemokines and ED, and further research is required to elucidate whether these cytokines/chemokines act solely as biomarkers as evidenced by our results or might play a causal role in ED. Notably, the two populations of rats at day 90, with and without ED, had no significant differences in disease severity and joint damage, suggesting that the levels of systemic pro-inflammatory cytokines are more predictive of ED than the severity of the disease. Consistent with this, a recent study reported that changes in disease activity (DAS28 score) did not correlate with endothelial function in RA patients [26].

At present, efforts to characterize endothelial function by measuring soluble plasma biomarkers in RA patients have been largely unsuccessful. Strikingly, albeit a role for inflammation in arthritis-induced ED is highly suspected, reports on the association between systemic inflammation and ED in RA patients are inconsistent. A positive relationship between circulating markers of inflammation and ED was reported by some authors [27–29] whereas others showed no associations both in early [30] and long-term RA [11]. In the present study, we demonstrated that plasma levels of IL-1β and TNF-α correlated with ED throughout the arthritis course. Of interest plasma levels of MIP-1α also correlated with ED. MIP-1α is a chemotactic factor for monocytes and T lymphocytes mainly secreted by synoviocytes in RA [31]. To the best of our knowledge, while high MIP-1α levels have been reported in RA patients [31], its potential role on ED has never been explored and warrants further investigation. Our data revealed that plasma levels of MCP-1 did not correlate with ED, in agreement with the findings of Södergen [27] in RA patients. These data are not consistent with a recent study conducted in collagen-induced arthritis in mice (mCIA) [32] in which an inverse correlation between serum MCP-1 levels and Ach-induced vasodilation was found. However, the study was performed only at the time of maximal clinical inflammation and whether MCP-1 levels reflected ED during the whole course of mCIA was not investigated. Emerging data highlighted OPG as a new promising biomarker of ED in various diseases [33, 34]. This protein of utmost importance for bone remodelling is expressed in other tissues including vessels [35]. In our study, no association was found between OPG and endothelial function in AIA. A few studies in RA patients reported an association between high plasma OPG levels and indices of carotid atherosclerosis [36, 37]. Our results encourage performing further studies to investigate the link between OPG levels and endothelial *function* in RA.

The present study demonstrated that modifications of endothelial phenotype occur at the first clinical symptoms of arthritis. However, a few clinical studies demonstrated that increased CV risk precedes RA onset [38, 39]. In our study, the unique detectable vascular dysfunction at the preclinical stage was a depressed contractile response to NE without alteration of the contractile response to KCl. As this finding was obtained in endothelium-denuded rings, the problem relies on VSMCs and not on endothelial cells. Our result obtained in AIA rats resonates with the recent finding of Reynolds et al [40] showing impaired contractile response to serotonin in the very early stage of development of mCIA that was, like in our study, unrelated to endothelial function. We added the new information that this contractile dysfunction is only transient during the course of arthritis. At present it is difficult to speculate on the underlying mechanisms. A role of pro-inflammatory cytokines can be discarded because TNF-α and IL-1β levels are not elevated at this time of arthritis. From a clinical perspective, our data give arguments to focus interest on the vascular contractile response for an early diagnostic of vascular dysfunction in RA, using for instance “low-flow mediated constriction” (L-FMC), a new technique introduced to assess in humans the conduit artery constrictor response to decreased flow [41].

In conclusion, the present study in AIA rats provided new insights in the mechanisms of ED in RA, and showed that a dysregulation of vasodilator and vasoconstrictor responses occurs with a distinct time course in RA progression. From a diagnostic perspective, they identified plasma IL-1β, TNF- α and MIP-1α as promising biomarkers of ED in RA. From a therapeutic perspective, they emphasized a seminal role for the cross-talk between NOS and COX-2 in RA-associated ED that encourage investigating the potential of new drugs such as NO-releasing NSAIDs [42] to decrease CV risk in RA.
