# Correction: Endothelial Dysfunction in Rheumatoid Arthritis: Mechanistic Insights and Correlation with Circulating Markers of Systemic Inflammation

**DOI:** 10.1371/journal.pone.0150874

**Published:** 2016-03-01

**Authors:** The PLOS ONE Staff

The image for Fig 4 is incorrect. The publisher apologizes for the error. Please see the corrected Fig 4 here.

**Fig 4:**
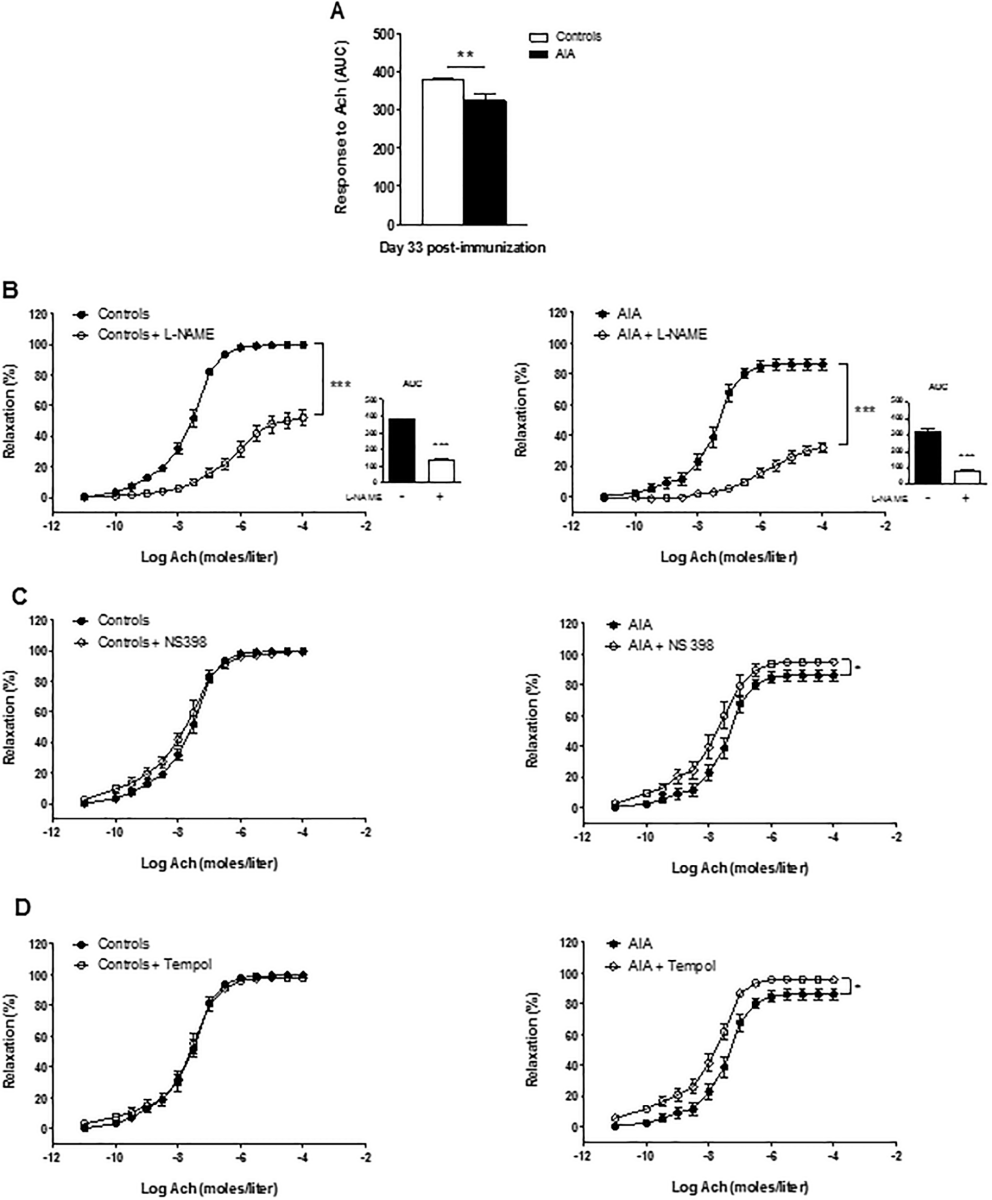
Effect of L-NAME, NS398 and Tempol on response to Ach at the acute inflammatory phase of AIA. (A) The vasodilator response to Ach (expressed as AUC of Ach) was assessed in aortic rings from AIA and controls at day 33 post-immunization. The same experiment was performed after incubation with L-NAME (B) (the insert presents AUC of Ach), NS398 (C) and Tempol (D). Results are expressed as means ± SEM of 12 to 19 aortic rings from 19 rats/group. ** p<0.01, *** p<0.001.
